# A Qualitative Study of HIV Testing Experiences and HIV Self-Testing Perspectives among Men in Northern Nigeria

**DOI:** 10.1155/2024/8810141

**Published:** 2024-04-20

**Authors:** Zubairu Iliyasu, Zikrillahi A. Haladu, Bilkisu Z. Iliyasu, Aminatu A. Kwaku, Nafisa S. Nass, Taiwo G. Amole, Hadiza M. Abdullahi, Amina U. Abdullahi, Fatimah I. Tsiga-Ahmed, Auwal Abdullahi, Humayra A. Bashir, Hamisu M. Salihu, Muktar H. Aliyu

**Affiliations:** ^1^Department of Community Medicine, Bayero University, Kano, Nigeria; ^2^VBRCH Training Program, Aminu Kano Teaching Hospital, Kano, Nigeria; ^3^Kano Independent Research Centre Trust, Kano, Nigeria; ^4^Vanderbilt Institute for Global Health, Vanderbilt University Medical Center, Nashville, TN, USA

## Abstract

HIV self-testing (HIVST) holds promise for accessing hard-to-reach populations by overcoming sociocultural and structural barriers to awareness of HIV status. This phenomenological qualitative study explored the experiences and perspectives of married men in Kano, northern Nigeria, regarding HIV testing and counseling (HTC) and HIVST. Twenty married men from diverse socioeconomic backgrounds participated in in-depth interviews conducted in the local language. Thematic analysis was employed to analyze the data, yielding key themes related to prior test experiences, knowledge of self-testing, and perceived ease of use, in addition to motivation for self-testing and concerns about reliability and counseling support. The findings shed light on the impact of facility-based HIV testing experiences on the perspectives of participants. Concerns related to delays, overcrowding, discomfort, fear, and unsupportive attitudes from healthcare providers influenced their perceptions. Among persons with previous self-testing experience, initial uneasiness was overcome with repeated use, highlighting the ease of use associated with HIVST. Motivations for self-testing included privacy, convenience, personal empowerment, improved infection detection, and efficiency. Concerns were raised regarding the reliability of self-testing results compared to hospital-based testing, and the absence of counseling support during self-testing. Our findings underscore the need to address infrastructural limitations, enhance counseling support, and promote awareness and knowledge of HIVST.

## 1. Introduction

Africa bears a disproportionate burden of the HIV pandemic, with approximately two-thirds of the world's 39 million people living with HIV residing in the region [1]. Despite programmatic efforts at scaling up HIV testing and counseling (HTC) using various strategies, a significant proportion of individuals living with HIV (approximately 14%) are still unaware of their status, leading to missed opportunities for prevention, treatment, and supportive care [1], and hindering progress towards the UNAIDS 95-95-95 targets.

Married men play a crucial role in shaping the health behaviors of their partners and families [2]. However, effectively reaching them with conventional HTC services remains challenging due to multiple factors operating at individual, sociocultural, and structural levels. At the individual level, factors such as fear of a positive result, denial, and perceived low risk contribute to men's reluctance to undergo HTC [3]. Similarly, sociocultural influences, including traditional gender norms and stigma surrounding HIV/AIDS, often discourage men from seeking testing services [4]. Moreover, structural barriers such as limited access to testing facilities, lack of targeted outreach, and inconvenient operating hours hinder men's engagement with HTC [5]. Given these multiple barriers, innovative strategies and tailored interventions are necessary to effectively reach and engage married men in HIV testing programs.

HIV self-testing (HIVST) has emerged as a promising approach to expanding access to HIV testing and improving early detection rates worldwide [6]. By allowing individuals to test for HIV in the privacy of their own homes, HIVST offers convenience and reduces barriers associated with facility-based testing, such as stigma, long waiting times, and limited access to healthcare facilities [7]. Understanding the perspectives of married men regarding HIVST is crucial for developing culturally appropriate interventions that effectively address their specific needs and preferences. This understanding can increase the uptake of HIV testing among married men and ultimately enhance HIV prevention, treatment, and control efforts.

In Nigeria, the landscape of HIV testing and counseling is characterized by a mix of facility-based and community-based approaches, including standalone HTC centers, mobile testing units, and integrated testing services offered in healthcare facilities [8]. Despite these efforts, disparities in access to HIV testing persist, particularly among men and in rural areas [9]. Men often face challenges accessing HTC services due to factors such as distance to testing sites, fear of stigma, and concerns about confidentiality [10]. In addition, cultural beliefs and gender norms may discourage men from seeking testing, perpetuating the cycle of undiagnosed HIV infection [11].

The present study aims to address this gap by exploring the experiences and perspectives of married men in Kano, northern Nigeria, regarding HIV testing and counseling (HTC) and HIV self-testing (HIVST). Through in-depth interviews, we seek to understand the factors influencing married men's decisions regarding HIV testing, including their experiences with existing testing modalities, barriers to testing, and perceptions of HIVST. By shedding light on the current landscape of HTC in the local setting and elucidating the motivations and concerns of married men, this study aims to inform the development of targeted interventions to improve HIV testing uptake and promote early detection of HIV infection among this key population.

## 2. Materials and Methods

We employed a phenomenological qualitative research design to gain insights into previous HIV testing experiences and explore the perspectives of married men regarding HIV self-testing (HIVST). Phenomenology was chosen as the research design to enable a comprehensive understanding of participants' lived experiences, attitudes, and beliefs, capturing rich and nuanced data.

Ethical approval for this study was obtained from the Kano State Ministry of Health Research Ethics Committee. Informed consent was obtained from all participants. Participants were provided with a detailed explanation of the purpose of the study, procedures, potential risks, benefits, and their rights as participants. They were assured of their confidentiality and the anonymity of their responses. Written consent was obtained from each participant, and they were informed of their right to withdraw from the study at any time without repercussions.

The sample size of 20 participants was determined based on the principles of qualitative research, aiming for an in-depth exploration of married men's experiences and perspectives on HIV testing and self-testing, ensuring diversity across socioeconomic backgrounds, marital status, and other relevant factors, allowing for data saturation and effective exploration of research objectives [12]. A purposive sampling approach was employed to select 20 married men from diverse socioeconomic backgrounds in Tarauni and Kumbotso local government areas in Kano, Nigeria. This approach ensured the inclusion of participants with a range of perspectives and experiences [13]. Through purposive sampling, the researchers identified men in monogamous or polygynous marriages who had undergone HIV testing and counseling (HTC), as well as those who had not. The participants were selected based on their varying levels of education, occupation, religion, ethnicity, income levels, and their knowledge of experience with HIVST. This selection strategy aimed to gather valuable insights that would address the research questions and objectives effectively.

In-depth interviews were conducted as the primary data collection method [14]. Experienced qualitative interviewers proficient in the local Hausa language conducted the interviews, establishing a rapport with the participants and creating an environment conducive to open and honest dialogue [15]. Conducting the interviews in the local language ensured that participants felt comfortable expressing their thoughts and experiences fully. Each interview lasted approximately 45–60 minutes, allowing participants ample time to share their perspectives in detail (see Supplemental file for interview script).

To ensure accuracy and facilitate data analysis, the interviews were audio-recorded and transcribed verbatim [16]. The transcripts and field notes recording nonverbal cues served as the foundation for subsequent analysis and interpretation. The researchers meticulously transcribed the interviews, capturing both the explicit content and the nuanced aspects of participants' responses. NVivo (version 13, QSR International) was used for qualitative data analysis.

Thematic analysis was employed as the analytical framework for this study [12, 17]. Initially, the researchers familiarized themselves through immersion in the data by repeatedly reading the transcripts, gaining a holistic understanding of the content. Subsequently, the researchers engaged in coding, systematically labeling segments of the data related to specific concepts or themes.

Throughout the coding process, codes were further categorized and organized into themes, enabling the identification of commonalities and patterns within the data [18]. This iterative analysis approach involved constant comparison, where the researchers examined similarities and differences across interviews, refining the emerging themes accordingly.

The thematic analysis approach used in this study was primarily inductive. Through immersion in the data, the researchers systematically identified patterns, concepts, and themes directly from the participants' narratives, without imposing preconceived theoretical frameworks or predetermined codes. The emergent themes were derived directly from the data, allowing for a comprehensive exploration of participants' experiences, attitudes, and beliefs regarding HIV testing and self-testing.

To enhance the rigor and credibility of the findings, the research team engaged in discussions and debriefing throughout the analysis process [15]. These discussions facilitated peer debriefing and exploration of alternative interpretations, ensuring the reliability and validity of the findings. Interviews were conducted by ZI, ZH, and AA, transcriptions were carried out by BI, AK, NN, and TA, and analysis was performed by ZI, ZH, HB, HS, and MA.

## 3. Results

A total of 20 married men participated in in-depth interviews. The average age of the participants was 42 years, with ages ranging from 30 to 69 years. The participants were evenly distributed between urban and rural areas, with half residing in each setting. Regarding marital status, nine men were in polygynous marriages. In terms of education, 14 participants had completed postsecondary education, while six had completed secondary education. A total of 11 participants were civil servants, four were businessmen, and five were self-employed. All participants in the study were adherents of the Islamic religion and of Hausa or Fulani ethnicity (Table 1).

The in-depth interviews revealed several key themes, including participants' prior test experiences, limited knowledge of self-testing, motivations for self-testing, perceived ease of use, demotivators, and concerns about reliability and counseling support (Table 2).

### 3.1. Theme: Prior HIV Test Experience

Participants who had prior HIV counseling and testing did so as preadmission requirements to educational institutions, preemployment, premarital requirements, or based on health provider advice. They described their experiences at public hospitals. Several participants expressed concerns about overcrowding, long queues, and delayed test results. One participant described his experience as follows:

*“My experience was terrible. I went to the testing center within the government hospital early in the morning, but I met a long queue. When it came to my turn after a long wait, the blood sample was taken. But, before the test result came out I was so scared that it might come out positive. What I disliked most was the long wait before the result was disclosed to me.”* (Businessman, 53 years)

Another participant highlighted his discomfort with being seen at a testing center and assumed to be HIV positive.

*“There was a large crowd with long queues and you know being at a testing center, you don't want to be seen in such places. I was afraid that someone who knows me will recognize me and spread the word that they saw me at a HIV test center. The fear of being labeled HIV-positive made me uncomfortable.”* (Civil servant, 34 years)

### 3.2. Theme: Concerns and Challenges with the Testing Process

Some participants expressed concerns related to the HIV testing process itself. These included fear or anxiety about a HIV-positive status, particularly for first-time testers, discomfort or pain from the needle prick, and nonsupportive attitudes from certain healthcare providers.

*“Well, the fear is there, and this is what makes us human… I hope you understand this feeling. Another thing I disliked about the testing process was the painful pricking to draw blood, either from the finger or vein. As a first-time tester, I had a lot of trouble with it.”* (Civil servant, 32 years)

Apart from fear of the outcome, another participant highlighted the lack of support from healthcare providers.

*“For me, it was the fear and psychological destabilization I experienced prior to knowing the test outcome. When you are about to collect the result, there is a deep-seated fear, numbness, and imbalance within you. Moreover, the healthcare workers were not as accommodating, offering no counseling, no smiles, and looking at you with a judgmental expression.”* (Businessman, 39 years)

### 3.3. Theme: Diverse Opinions on the Testing Process

Opinions on the HIV testing process varied among participants. While some participants found the process to be smooth, straightforward, and simple, others had negative experiences and encountered challenges. Several participants emphasized the importance and value of pretest and posttest counseling as positive aspects of their testing experience. A participant shared a good experience, attributing it to the pretest counseling he received as follows:

*“I can say my experience was good because there was pretest counseling during which the health personnel assured me that even if my test result came out positive, there are medications available. I found the health workers welcoming.”* (Civil servant, 34 years)

Another participant highlighted his initial discomfort which gave way to relief and a sense of freedom.

*“I was not comfortable prior to the test, but immediately after the smooth testing process and being declared negative, I felt free and comfortable. What I liked most was the fact that the process was smooth, simple, and straightforward and fortunately for me the result came out negative.”* (Businessman, 58 years)

Among those who did not undergo HTC, their reasons revolved around trusting themselves and their marital partners. One participant expressed this sentiment, stating that

*“No, I have never tested for HIV. Why should I? There is no need. I am healthy, and when it comes to any illness related to sexual activity, it doesn't even cross my mind because I know I am healthy. I trust my spouse, and I only engage in sexual intercourse with my wife. Therefore, I don't even consider getting an HIV test.”* (Self-employed, 65 years)

### 3.4. Theme: Limited Knowledge of HIV Self-Testing

Most participants were unaware of HIV self-testing (HIVST) prior to the interviews. They expressed surprise upon learning about the availability of a HIV test that could be conducted outside of a clinical setting. Some were particularly intrigued by the use of saliva instead of a blood sample during self-testing.

When asked if they had heard about rapid HIVST before the study, typical responses included the following:

*“No, I haven't. Today is the first-time I am hearing about it. I only know of the one they do in the hospital by drawing blood from the finger. So I thought it would be that one.”* (Businessman, 58 years)

*“I never knew a person can self-test on their own prior to this study. I thought HIV tests could only be done in a hospital or laboratory setting by doctors and other health workers. Actually, today is the first-time I'm hearing about it.”* (Self-employed, 50 years)

A few participants had prior knowledge of HIVST through various sources such as television, radio, friends, or healthcare providers.

One participant shared his initial skepticism and subsequent discovery as follows:

*“I heard about it on a radio program and commercials, but I didn't think it was real because I was thinking, how is it possible to do a HIV test at home? How can someone interpret and report the result? Later, I browsed the internet and was surprised to find out that it really exists.”* (Civil servant, 34 years)

### 3.5. Theme: Perceived Advantages of Self-Testing

#### 3.5.1. Ease of Use

Participants who had previous experience with self-testing initially felt uneasy or apprehensive when using HIVST for the first time. However, as they gained experience, they became more comfortable and confident in independently conducting the test. They expressed assurance in their ability to follow the instructions without supervision or guidance.

*“For my first self-testing experience, I was overwhelmed and nervous about whether I could perform the test correctly. However, after watching a video demonstration on how to do the test, it became easy and straightforward.”* (Civil servant, 53 years)

*“I felt good using HIV self-testing. It was easy to use, and I didn't have to spend money on transportation to go to the hospital. It also provided confidentiality. I used the oral test, and it was easy to perform. The only difficulty I faced was in following the instructions.”* (Businessman, 58 years)

#### 3.5.2. Motivation to Self-Test for HIV

Participants expressed motivations for engaging in HIV self-testing (HIVST) that revolved around independence and greater privacy, in contrast with healthcare facility settings where others are present. They also viewed HIVST as a source of empowerment.

*“First of all, HIV self-testing gives one the flexibility to perform the test whenever needed. Secondly, it ensures confidentiality about one's health outcome, as it is not exposed to others. Thirdly, it provides individuals with absolute confidence in knowing their health status.”* (Civil servant, 32 years)

*“Testing in public can discourage people from getting tested due to the possibility of others looking at you and making assumptions. But with self-testing, it is completely private, saves time and cost by eliminating transportation and hospital charges.”* (Married man, 60 years)

Participants highly valued the privacy and confidentiality of self-testing. They perceived clinic visits, especially when the visibility of HIV testing and counseling (HTC) rooms allowed others to observe, as potential invasions of privacy.

*“The idea of self-testing motivates me because, in a clinic, people already know what is happening in the specific room you are entering. So, when you come out, they stare at you, trying to interpret your facial expression. But with HIV self-testing, one can carry out the test in their own space, where no one is watching.”* (Self-employed, 50 years)

#### 3.5.3. Convenience and Efficiency

Participants also highlighted the convenience and efficiency of HIV self-testing as motivating factors. They recognized the opportunity to test themselves and their family members in the comfort of their homes. The freedom to perform the test at a chosen time and place, without the need for a clinic visit, was viewed as advantageous and timesaving.

*“It is convenient to perform the test and to discover one's HIV status early, allowing for early medication initiation. Many people do not want to go to the hospital and wait in long queues, especially men. HIV self-testing bypasses those issues and prevents time wasted in queues.”* (Businessman, 57 years)

#### 3.5.4. Personal Empowerment

Participants recognized that having a self-test kit empowered them to proactively monitor their HIV status, fostering a sense of personal responsibility for their health.

*“It puts one in charge and in control of their health, enabling them to adjust their behavior accordingly. It saves time and enhances interest in protecting one's health status.”* (Civil servant, 48 years)

#### 3.5.5. Increased Detection of Infection

Participants believed that HIVST is beneficial for increasing the detection of infections, leading to early treatment initiation and improved outcomes. They recognized the advantages of HIVST and appreciated its merits.

*“I would often tell myself that I will go to the hospital on a certain date to test, but in the end, I couldn't. Doing the HIV self-testing on my own would be easier, and it would enable not only me but also others to detect the infection early and seek treatment before it's too late.”* (Self-employed, 58 years)

#### 3.5.6. Ease of Use and Speed

Participants emphasized the advantages of HIVST, including convenience, ease of use, and speed. These factors served as key motivators, emphasizing the convenience and efficiency provided by HIVST.

*“We can easily do the test because saliva is in our mouth, whereas drawing blood might be scary for some people. It's quick, can be done anywhere, and involves only a little swab. There's even no blood involved.”* (Businessman, 53)

*“It's quick. You can do it anywhere. You must go by yourself to a place like a pharmacy. It's only a little swab. There's no blood involved.”* (Civil servant, 46 years)

#### 3.5.7. Time-Saving Features

HIVST addresses the concerns of married men by eliminating long queues, fear of recognition, and discomfort in testing centers. Participants indicated that HIVST bypasses issues associated with facility-based HIV testing and counseling (HTC), saving time for individuals.

### 3.6. Theme: Demotivators/Reasons for Avoiding HIV Self-Testing

Participants mentioned several reasons for not engaging in HIV self-testing. The first was a lack of awareness about the availability of HIV self-tests. They expressed that they could not engage in something they were unaware of.

*“I never knew there is a test for HIV that a person can do at home. So, you see, I cannot do what I don't know.”* (Businessman, 53 years)

The second reason was concerns about not having a medical professional present at home in case of a positive diagnosis, particularly for individuals with underlying mental illnesses. They highlighted the need for professional guidance and support during such situations.

*“Let's say a person is mentally ill, suffering from depression or something, and they happen to test positive at home. They would have nobody to talk to at that point in time. They might act impulsively without the guidance of a trained medical professional. So, you see, someone scary might happen.”* (Civil servant, 34 years)

The third reason was concern about the reliability of results obtained through HIVST compared to hospital-based testing. They emphasized the importance of accuracy and reliability in HIV testing, particularly in ensuring trustworthy results from self-testing kits.

*“Firstly, there is the issue of reliability. If I do it myself, I am not a health professional. Presenting the test result to certain organizations, the result may not be considered valid and as a normal hospital test. Additionally, the test requires training. To get an accurate result I would have to be trained before testing myself.”* (Civil servant, 46 years)

*“The HIV self-test result may be a false negative or false positive result, unlike the test done in the hospital, which I believe is more reliable. And since I am not a medical professional, there is a chance that I may miss some steps or not perform the procedure correctly as professionals would. This could invalidate the test result.”* (Civil servant, 62 years)

### 3.7. Theme: Lack of Counseling and Psychological Support

Participants were worried about the absence of counseling during the self-testing process and the potential risk of emotional distress, including the risk of suicide, among individuals who received a positive HIV test result without pretest counseling. Some individuals may not be emotionally prepared and lack the necessary support systems to cope with a positive result.

*“There are many disadvantages. To me, they include the absence of a counselor during testing and the mistakes that could occur while performing or interpreting the test result. The test that a doctor or health care professional would perform is more accurate than a self-test.”* (Civil servant, 33 years)

*“I am worried about the lack of counseling with the self-test. There should be counseling, even virtually and if someone tests positive, they should be enrolled to see a doctor for appropriate care.”* (Civil servant, 51 years)

Participants recognized the value of counseling in HIV self-testing, which need not be extensive and could be online or in the form of helplines. They emphasized the need for the provision of accurate information and guidance on accessing appropriate care and support in the event of a positive result.

*“One downside of self-testing is the lack of counseling and knowledge about HIV. The self-test can be a big deal if you do not have counseling and information. Therefore, pre- and post-test counseling should be provided.”* (Self-employed, 65 years)

### 3.8. Theme: Reduced Chance of Disclosure

Some participants perceived a reduced likelihood of seeking care among those who test positive through self-testing because there will not be any external pressure, as they are the only ones aware of the positive result. This was considered a significant disadvantage or demotivator for HIVST. They emphasized the importance of a seamless integration between self-testing and timely linkage to the continuum of HIV care.

*“It will bring trouble. Number one is that a person can keep the positive result to themselves, not telling anyone. So, there will be no linkage to treatment and prevention services. This can lead to the spread of the disease to many people.”* (Civil servant, 33 years)

*“Someone may decide to keep quiet after testing positive and not seek care in a hospital.”* (Businessman, 43 years)

## 4. Discussion

This qualitative study aimed to explore the experiences, motivations, and perceptions of HIV testing among married men in northern Nigeria, with a specific focus on HIV self-testing (HIVST). The analysis of the data revealed several key themes, including participants' prior experiences with HIV testing, limited knowledge of self-testing, motivators, and demotivators for self-testing, perceptions of the ease of use of self-testing, and concerns about its reliability and the availability of counseling support.

The participants' previous experiences with facility-based HIV counseling and testing emerged as a crucial factor influencing their perspectives. These experiences often involved delays, overcrowding, and long waiting times, consistent with previous reports [14–17]. These findings highlight the urgent need to address the inefficiency and suboptimal quality of facility-based testing services. Measures must be taken to alleviate the obstacles that discourage individuals from seeking HIV testing, including fear of stigmatization or being recognized by others at test centers. Negative experiences during testing can undermine the overall testing process, leading to reluctance for future testing and recommendations to others. One potential solution is the implementation of a prebooked appointment system.

Participants expressed concerns about the testing process itself, including discomfort, fear, and nonsupportive attitudes of healthcare providers, which align with previous research [18–21]. These findings highlight the importance of providing interpersonal communication skills training to healthcare providers and creating a supportive and compassionate environment in HIV testing centers to promote regular testing, early detection, and linkage to treatment.

Opinions on the HIV testing process varied among participants, with some describing negative experiences, while others found it to be smooth and simple. These diverse perspectives emphasize the need for standardized protocols, quality assurance measures, and integrating counseling services to ensure consistency in testing procedures and timely disclosure of results across different testing sites. Moreover, integrating counseling services with the testing process is crucial to addressing the psychological and emotional needs of individuals, providing accurate information and offering appropriate support before and after testing.

The participants' lack of knowledge and awareness about self-testing was evident, although some had heard about it through the media or healthcare providers. This highlights the potential role of information dissemination and health education in increasing awareness and knowledge about HIV self-testing, consistent with previous research [22, 23]. Utilizing various media platforms can be effective in disseminating information about HIVST and its advantages.

The motivations for HIV self-testing in this study, including privacy, convenience, personal empowerment, and increased detection of infection, are consistent with previous research findings [7, 22]. These findings highlight the importance of these motivations in shaping individuals' preferences for self-testing and align with the principles of patient-centered care and autonomy.

Overcoming initial apprehension about self-testing and gaining increased confidence through repeated testing suggest that individuals can adapt and overcome the challenges associated with self-testing. These findings align with previous studies that have investigated the ease of use and user experience of HIVST [24, 25]. To further enhance the uptake of HIVST, early adopters could be trained as peer educators to promote self-testing and provide support to their communities.

The lack of immediate access to counseling during self-testing, as voiced by the participants, aligns with previous reports among different populations [26, 27]. Addressing this concern requires integrating counseling services, remote helplines, and offering supervised and unsupervised self-testing options.

The implications of this study for policy and practice include improving facility-based testing infrastructure, training healthcare providers in communication skills and patient-centered care, implementing standardized protocols, and increasing awareness through educational campaigns. Integrating counseling services, remote helplines, and offering supervised and unsupervised self-testing options can address concerns and enhance the acceptability and effectiveness of self-testing initiatives.

The richness and depth of the data enhance the credibility of the findings, providing valuable evidence for targeted interventions to improve HIV testing awareness among married men. However, it is important to recognize the limitations of qualitative studies in terms of generalizability. Future studies can explore HIV testing experiences among different demographic groups and in diverse cultural contexts. Mixed methods research designs can provide a more comprehensive understanding of the topic by combining qualitative and quantitative approaches.

## 5. Conclusion

This study underscores the importance of addressing concerns related to the testing process, improving access to testing, and creating a supportive environment for individuals seeking HIV testing. The motivations expressed for self-testing emphasize the need for privacy, convenience, personal empowerment, and increased detection of infections. Integrating counseling services and remote helplines and offering supervised and unsupervised self-testing options can enhance the effectiveness of self-testing programs. By implementing these recommendations, policymakers and healthcare professionals can contribute to increased testing uptake, early detection, and linkage to care, ultimately helping to end the HIV epidemic.

## Figures and Tables

**Table 1:** Demographic attributes of married men who participated in the in-depth interviews in Kano, Nigeria (*n* = 20).

Characteristics	Frequency
*Age (years)*	
30–39	6
40–49	4
50–59	8
≥60	2
*Residence*	
Rural	10
Urban	10
*Marriage type*	
Monogamous	11
Polygynous	9
*Level of education*	
Secondary and lower	6
Postsecondary	14
*Occupation*	
Self-employed	5
Businessman	4
Civil servant	11
*Religion*	
Islam	20
Other faiths	0

**Table 2:** Summary of themes, subthemes, and illustrative quotes.

Theme	Subtheme	Illustrative quote
Prior HIV test experience	Long wait times and overcrowding	“My experience was terrible. What I disliked most was the long wait before the result was disclosed to me.” (Businessman, 53 years)
	Fear of stigma associated with testing	“The fear of being labeled HIV-positive made me uncomfortable.” (Civil servant, 34 years)

Concerns and challenges with testing	Fear of a positive result	“Well, the fear is there. As a first-time tester, I had a lot of trouble with it.” (Civil servant, 32 years)
	Lack of supportive attitudes from providers	“The healthcare workers were not as accommodating, offering no counseling, no smiles.” (Businessman, 39 years)

Diverse opinions on the testing process	Positive experiences with counseling	“I can say my experience was good. I found the health workers welcoming.” (Civil servant, 34 years)
	Discomfort and fear of stigma	“I was not comfortable prior to the test fortunately for me the result came out negative.” (Businessman, 58 years)

Limited knowledge of self-testing	Surprise upon learning about self-testing	“Today is the first-time I am hearing about it.” (Businessman, 58 years)
	Initial skepticism	“I didn't think it was real. Later, I browsed the Internet and was surprised to find out that it really exists.” (Civil servant, 34 years)

Perceived advantages of self-testing	Ease of use	“For my first self-testing experience, I was overwhelmed and nervous, it became easy and straightforward.” (Civil servant, 53 years)
	Motivation for self-testing	“HIV self-testing gives one the flexibility and provides individuals with absolute confidence in knowing their health status.” (Civil servant, 32 years)
	Privacy and confidentiality	“The idea of self-testing motivates me because one can carry out the test in their own space.” (Self-employed, 50 years)
	Convenience and efficiency	“It is convenient to perform the test. HIVST bypasses those issues and prevents time wasted in queues.” (Businessman, 57 years)
	Personal empowerment	“It puts one in charge and in control of their health enhances interest in protecting one's health status.” (Civil servant, 48 years)
	Increased detection of infection	“Doing the HIVST on my own would be easier to detect the infection early and seek treatment before it's too late.” (Self-employed, 58 years)
	Ease of use and speed	“It's quick, can be done anywhere. There's even no blood involved.” (Businessman, 53 years)

Demotivators/reasons for avoidance	Lack of awareness	“I never knew there is a test for HIV that a person can do at home.” (Businessman, 53 years)
	Concerns about lack of professional support	“They might act impulsively without the guidance of a trained medical professional.” (Civil servant, 34 years)
	Concerns about the reliability of self-testing	“The test result may not be considered valid could invalidate the test result.” (Civil servant, 46 years)

Lack of counseling and support	Worries about emotional distress	“There are many disadvantages. The test that a doctor or health care professional would perform is more accurate than a self-test.” (Civil servant, 33 years)
	Recognition of the value of counseling	“One downside of self-testing is the lack of counseling pretest and posttest counseling should be provided.” (Self-employed, 65 years)

Reduced chance of disclosure	Perception of reduced likelihood of seeking care	“There will be no linkage to treatment and prevention services. This can lead to the spread of the disease to many people.” (Civil servant, 33 years)

## Data Availability

The data used to support the findings of the study are available from the corresponding author upon request and in strict accordance with the data privacy rules as set forth by the government of Nigeria.

## Supplementary Materials

Supplementary Materials Interviewer guide/script for participant interviews.

## References

[1] Unaids World Aids Day (2023). Fact sheet. Global HIV statistics.

[2] Matovu J. K. B., Kisa R., Buregyeya E. (2018). ’If I had not taken it [HIVST kit] home, my husband would not have come to the facility to test for HIV’: HIV self-testing perceptions, delivery strategies, and post-test experiences among pregnant women and their male partners in Central Uganda. *Global Health Action*.

[3] Jones J., Carter B., Wilkerson R., Kramer C. (2019). Attitudes toward HIV testing, awareness of HIV campaigns, and using social networking sites to deliver HIV testing messages in the age of social media: a qualitative study of young black men. *Health Education Research*.

[4] Camlin C. S., Ssemmondo E., Chamie G. (2016). SEARCH Collaboration. Men missing from population-based HIV testing: insights from qualitative research. *AIDS Care*.

[5] Tshuma N., Muloongo K., Setswe G. (2015). Potential barriers to rapid testing for human immunodeficiency virus among a commuter population in Johannesburg, South Africa. *HIV/AIDS (Auckland, N.Z.)*.

[6] World Health Organization (2023). *Guidelines on HIV Self-Testing and Partner Notification: Supplement to Consolidated Guidelines on HIV Testing Services*.

[7] Bwalya C., Simwinga M., Hensen B. (2020). The HPTN 071 PopART study team Social response to the delivery of HIV self-testing in households: experiences from four Zambian HPTN 071 (PopART) urban communities. *AIDS Research and Therapy*.

[8] Ahmed S., Delaney K., Villalba-Diebold P. (2013). HIV counseling and testing and access-to-care needs of populations most-at-risk for HIV in Nigeria. *AIDS Care*.

[9] Sibanda E. L., Taegtmeyer M. (2020). Inequalities in uptake of HIV testing despite scale-up. *Lancet Global Health*.

[10] Dirisu O., Eluwa G., Adams E., Torpey K., Shittu O., Adebajo S. (2020). I think this is the only challenge… the stigma Stakeholder perceptions about barriers to Antenatal care (ANC) and Prevention of mother-to-child transmission (PMTCT) uptake in Kano state, Nigeria. *PLoS One*.

[11] Durevall D., Lindskog A. (2015). Intimate partner violence and HIV in ten sub-Saharan African countries: what do the Demographic and Health Surveys tell us?. *Lancet Global Health*.

[12] Morse J. M. (2015). Data were saturated. *Qualitative Health Research*.

[13] Tongco M. D. C. (2007). Purposive sampling as a tool for informant selection. *Ethnobotany Research and Applications*.

[14] Tindall L., Smith J. A., Flower P., Larkin M. (2009). J.A. Smith, P. Flower and M. Larkin (2009), *Interpretative phenomenological analysis: theory, Method and research*.: London: sage. *Qualitative Research in Psychology*.

[15] Horsfall M., Eikelenboom M., Draisma S., Smit J. H. (2021). The effect of rapport on data quality in face-to-face interviews: beneficial or detrimental?. *International Journal of Environmental Research and Public Health*.

[16] McMullin C., Methods Q. (2023). Transcription and qualitative methods: implications for third sector research. *Voluntas*.

[17] Nowell L. S., Norris J. M., White D. E., Moules N. J. (2017). Thematic analysis: striving to meet the trustworthiness criteria. *International Journal of Qualitative Methods*.

[18] Neale J. (2016). Iterative categorization (IC): a systematic technique for analysing qualitative data. *Addiction*.

[19] Meehan S. A., Leon N., Naidoo P., Jennings K., Burger R., Beyers N. (2015). Availability and acceptability of HIV counselling and testing services. A qualitative study comparing clients’ experiences of accessing HIV testing at public sector primary health care facilities or non-governmental mobile services in Cape Town, South Africa. *BMC Public Health*.

[20] Meremo A., Mboya B., Ngilangwa D. (2016). Barriers to accessibility and utilization of HIV testing and counseling services in Tanzania: experience from Angaza Zaidi programme. *Pan Afr Med J*.

[21] Ngangue P., Gagnon M. P., Bedard E. (2017). Challenges in the delivery of public HIV testing and counselling (HTC) in Douala, Cameroon: providers perspectives and implications on quality of HTC services. *BMC International Health and Human Rights*.

[22] Mwamba C., Sharma A., Mukamba N. (2018). ’They care rudely!’: resourcing and relational health system factors that influence retention in care for people living with HIV in Zambia. *BMJ Global Health*.

[23] Ahmed S. I., Syed Sulaiman S. A., Hassali M. A., Thiruchelvam K., Hasan S. S., Lee C. K. (2017). Attitudes and barriers towards HIV screening: a qualitative study of people living with HIV/AIDS (PLWHA) in Malaysia. *Journal of Infection Prevention*.

[24] Rao A., Nagourney E. M., Chen V. H. (2021). Assessing attitudes to ED-based HIV testing: development of a short-structured survey instrument. *PLoS One*.

[25] Dapaah J. M. (2016). Attitudes and behaviours of health workers and the use of HIV/AIDS health care services. *Nursing Research and Practice*.

[26] Kiene S. M., Sileo K., Wanyenze R. K. (2015). Barriers to and acceptability of provider-initiated HIV testing and counselling and adopting HIV-prevention behaviours in rural Uganda: a qualitative study. *Journal of Health Psychology*.

[27] Tan Y. R., Kaur N., Ye A. J. (2021). Perceptions of an HIV self-testing intervention and its potential role in addressing the barriers to HIV testing among at-risk heterosexual men: a qualitative analysis. *Sexually Transmitted Infections*.

