# Supplementary material for: A Qualitative Study of HIV Testing Experiences and HIV Self-Testing Perspectives among Men in Northern Nigeria
Source: Nurs Res Pract. 2024 Apr 20;2024:8810141. doi: 10.1155/2024/8810141 (PMC11055649; doi:10.1155/2024/8810141)
Supplement: Supplementary Materials — Interviewer guide/script for participant interviews. [file 8810141.f1.docx]

**SUPPLEMENTAL FILE**

**Interview Guide/Script**

**Introduction**

Hello, I am …………………………, from the Department of Community Medicine at Bayero University Kano. We are conducting a study on the acceptability of HIV self-testing among married men in Kano.

**Interviewer:** *Obtain informed consent for interview and tape recording*

Before we proceed, I'd like to go through the Informed Consent Form with you. Your participation is voluntary, and your responses will be kept confidential. This interview will last for approximately 60 minutes, and your name or any identifying information will not be associated with your responses. The interview will be digitally audio-recorded, with your consent.

**Personal Information**

Participant ID: [ ] Interview date: Interviewer: Place of interview: Age: Gender: Education level: Occupation: Type of marriage: [Monogamy/Polygamy] Religion: LGA of Residence:

**Experiences with HIV-testing**

1. What comes to mind when you think about HIV testing?
2. Have you ever tested for HIV? a. For those who tested: Can you share your experience? b. For those who have NOT tested: Have you thought about testing before? If yes, where would you prefer to go?

**HIV Self-Testing (HIVST)**

1. Have you heard about HIV self-testing before?
2. What are your thoughts on testing yourself for HIV in private?
3. What motivates you to use HIV self-testing kits?
4. What are the drawbacks compared to HIV Counseling and Testing by a Health Care worker?
5. For those who have used HIVST:
   - How was your experience?
   - Why did you choose to use HIV self-tests instead of facility-based testing?

**Factors Influencing Acceptability**

1. What factors would influence your decision to use a self-test kit?
2. How much would you be willing to pay for a self-test kit?
3. Do you prefer testing yourself at home or in clinics? Why?
4. Where would you prefer to buy the self-test kits?
5. What support would you find most helpful before self-testing?
6. What factors would encourage you to confirm a positive result with a confirmatory test?

**Conclusion**

Thank you for your participation. Your responses are confidential, and your insights are valuable to our research. If you have any questions, feel free to ask. Once again, thank you for your time and cooperation.
